# Supplementary material for: Mapping Lymph Node during Indocyanine Green Fluorescence-Imaging Guided Gastric Oncologic Surgery: Current Applications and Future Directions
Source: Cancers (Basel). 2022 Oct 20;14(20):5143. doi: 10.3390/cancers14205143 (PMC9601265; doi:10.3390/cancers14205143)
Supplement: Supplementary file 1 [file cancers-14-05143-s001.zip › cancers-1968672-supplementary.pdf]

Table S1. Detailed implementation of SLN detection studies.

Ref	No. of Patients	Submucosal/ Subserosal	ICG Dosage (mg)	Waiting Time	Lymph Node (mean)	SLN (mean)	Near-Infrared System
Hiratsuka, M. 2001 [26]	74	subserosal	25	5 min	39	2.3	NA
Nimura, H. 2004 [36]	84	submucosal	2.5	20 min	NA	10.5	IREE (Olympus Optical, Tokyo, Japan)
Park, D. J. 2006 [25]	100	subserosal	25	5 min	37.3	4.4	NA
Kusano, M. 2008 [27]	22	subserosal	NA	NA	33.9	3.6	Infrared Camera System PDE (Hamamatsu Photonics, Hamamatsu, Japan)
Kelder, W. 2010 [28]	212	submucosal	2.5	20 min	NA	6	infrared ray electronic endoscopy (IREE, Olympus Optical, Tokyo, Japan)
Park, D. J. 2011 [29]	68	submucosal	NA	15 min	NA	2.5	NA
Miyashiro, I. 2013 [30]	241	submucosal/ subserosal	20–25	5 min	40	3.8	NA
Miyashiro, I. 2014 [38]	440	subserosal	25	NA	NA	4 (medium)	NA
Tummers, Q. R. 2016 [40]	22	subserosal	25	NA	24	4.4	Mini-FLARETM NIR fluorescence imaging system
Takahashi, N. 2017 [41]	44	submucosal	10	20 min	NA	4.1	IRLS (Olympus Optical, Tokyo, Japan) (infrared light observation)
Mayanagi, S. 2020 [42]	132	submucosal	NA	10-15 min	NA	5	NA
